# Anxiety and Cognition in Cre- Collagen Type II Sirt1 K/O Male Mice

**DOI:** 10.3389/fendo.2021.756909

**Published:** 2021-11-19

**Authors:** Biana Shtaif, Shay Henry Hornfeld, Michal Yackobovitch-Gavan, Moshe Phillip, Galia Gat-Yablonski

**Affiliations:** ^1^ Sackler School of Medicine, Tel Aviv University, Tel Aviv, Israel; ^2^ Laboratory for Molecular Endocrinology and Diabetes, Felsenstein Medical Research Center, Petach Tikva, Israel; ^3^ The Jesse Z and Sara Lea Shafer Institute for Endocrinology and Diabetes, National Center for Childhood Diabetes, Schneider Children’s Medical Center of Israel, Petach Tikva, Israel

**Keywords:** SIRT1, anxiety, cognition, collagen type II, osteocalcin

## Abstract

**Introduction:**

Using transgenic collagen type II-specific Sirt1 knockout (CKO) mice we studied the role of Sirt1 in nutritional induced catch up growth (CUG) and we found that these mice have a less organized growth plate and reduced efficiency of CUG. In addition, we noted that they weigh more than control (CTL) mice. Studying the reason for the increased weigh, we found differences in activity and brain function.

**Methods:**

Several tests for behavior and activity were used: open field; elevated plus maze, Morris water maze, and home cage running wheels. The level of Glu- osteocalcin, known to connect bone and brain function, was measured by Elisa; brain Sirt1 was analyzed by western blot.

**Results:**

We found that CKO mice had increased anxiety, with less spatial memory, learning capabilities and reduced activity in their home cages. No significant differences were found between CKO and CTL mice in Glu- osteocalcin levels; nor in the level of brain SIRT1.

**Discussion/Conclusion:**

Using transgenic collagen type II-specific Sirt1 knockout (CKO) mice we found a close connection between linear growth and brain function. Using a collagen type II derived system we affected a central regulatory mechanism leading to hypo activity, increased anxiety, and slower learning, without affecting circadian period. As children with idiopathic short stature are more likely to have lower IQ, with substantial deficits in working memory than healthy controls, the results of the current study suggest that SIRT1 may be the underlying factor connecting growth and brain function.

## 1 Introduction

In order to identify novel regulatory mechanisms in children’s growth, we have been focusing on the epiphyseal growth plate (EGP) and the important role of SIRT1 in regulating the nutrition-growth connection was unraveled (1, 2). The sirtuin (SIRT) family of proteins consists of proteins that act predominately as nicotinamide adenine dinucleotide-dependent (NAD+) – deacetylases and convey diverse functions in a variety of physiological settings.

SIRT1, the most conserved enzyme of this family and the most extensively studied, is a central component of numerous basic pathways. The expression and activity of SIRT1 are tightly regulated. SIRT1 is usually localized to the nucleus but may also translocate to the cytosol (3). It interacts and deacetylates histones and non-histone proteins such as p53, E2F1, NF-kB, and FOXO (4, 5) and through them affects cell proliferation, senescence, autophagy, stress responses, and apoptosis. SIRT1 has been shown to have a substantial role in longevity (6), response to food restriction (1, 7), and linear growth (1, 2).

We studied nutritional-induced catch-up growth (CUG) using a model consisting of food restriction followed by refeeding. SIRT1 was among the few proteins that was increased in EGPs of food-restricted rats (2). Additionally, we showed that miR-140 and miR-22 were reduced at the EGP by food restriction, and their mutual target, SIRT1, showed a subsequent increase (1). Next, we knocked out Sirt1 expression using a collagen-type II-specific Cre-Lox system (2), and investigated its role in nutrition-induced CUG.

Depletion of SIRT1 by using the collagen-II-specific Cre-Lox system led to increased height of the proliferative zone with less organized EGP. The CKO mice were less responsive to the nutritional manipulation, and their CUG was less efficient. They remained shorter than the CTL mice who corrected the food restriction-induced growth deficit during the re-feeding period. In addition, we identified a crucial role of SIRT1 in the response of the cortical and trabecular bone fractions to nutritional manipulation. Furthermore, there was a significant effect on animal weight, with the knockout mice being heavier than their control littermates. The difference in weight was more pronounced with age. Others showed that Sirt1+/− mice presented with early onset, moderate osteoarthritis relative to their wild type littermates (8). Therefore, we hypothesized that, as our model is cartilage specific, it may also affects articular cartilage and thus mobility. This led us to monitor the locomotor activity of the knockout mice in the open field, and thereafter to perform additional tests to examine effects on anxiety and spatial memory.

## 2 Materials and Methods

### 2.1 Animals

All the procedures and experiments were according to the Arrive guidelines (https://arriveguidelines.org) and approved by the Institutional Animal Care and Use Committee of Tel Aviv University, that follows the NIH guide for the care and use of laboratory animals (protocol approval number 01-16-052) prior to onset of the study. As most mice homozygous for the Sirt1-null allele do not survive for more than 1 month, we used the Cre-loxP system to knockout cartilage-specific Sirt1. Previous studies showed that crossing this floxed allele to a whole-body Cre-expressing mouse recapitulated the Sirt1-null phenotype (9). Sirt1-CKO (referred to throughout the manuscript as CKO) mice were generated as previously described (2); Col II-Cre-recombinase removes exon 4 of the floxed Sirt1 gene (encoding a catalytic domain of the protein) specifically in chondrocytes (2, 9). All mice were genotyped at the age of three weeks from tail snips (2). Male mice were chosen to eliminate the confounding factor of sex on linear growth. CKO mice were obtained at the expected Mendelian ratio, survived normally, and mated successfully. Sirt1 flox/flox littermate mice without the Cre transgene were used as controls (CTL). Mice were maintained under pathogen-free conditions (temperature controlled to 25 ± 1°C, humidity 50 ± 2%, 12h light/dark cycle (apart from the circadian rhythm study); lights off at 18:00h) and allowed free access to food (2018SC; Teklad Rodent Diet, Envigo, Madison, WI, USA) and tap water. The CKO and CTL mice showed no differences in size and weight at birth and no significant postnatal growth retardation. Mice were designated “young” when age was 4-8 months and “old” when age was >8-11 months.

### 2.2 The Open Field Test

In the open field test (OFT) mice were placed in a 47×47×51 cm arena for 30 min (CKO, n=37; CTL, n=32; **Table 1**). The total distance travelled and the duration of time exploring the periphery and the center of the arena were video-tracked under normal light conditions for 30 min using the EthoVision XT 11.5 software (EthoVision 3.1 Noldus Information Technology B.V., Wageningen, The Netherlands). Data was used to measure locomotor activity and to explore features indicative of greater anxiety-like behavior and decreased exploratory activity (10).

**Table 1:** Comparison between CKO mice and CTL mice in the Open Field test (OFT) according to age group (data is presented as mean ± SD).

	All	Young (<9 months)	Old (≥9 months)	Within group (P1)
**Table 1A (Open Field)**				
**N (number of measurements)**				
CKO	37	22	15	
CTL	32	22	10	
**Total distance (cm)**				
CKO	8468.9±2530.3	9103.5±2437.6	7487.0±2531.3	0.064
CTL	3824.1±891.2	4002.7±928.8	3437.4±692.0	0.097
Between groups (P2)	<0.001	<0.001	<0.001	
**Periphery (cm)**				
CKO	5670.3±1655.3	5893.0±1612.7	5305.9±1776.8	0.313
CTL	2407.4±713.5	2633.6±717.7	1909.8±389.3	0.006
Between groups (P2)	<0.001	<0.001	<0.001	
**Center (cm)**				
CKO	2791.0±1257.4	3205.9±1307.7	2173.2±946.5	0.015
CTL	1406.4±608.1	1351.6±671.8	1527.0±444.2	0.459
Between groups (P2)	<0.001	<0.001	0.037	
**Periphery (% of time)**				
CKO	76.1±9.0	74.5±8.3	77.9±10.1	0.294
CTL	68.0±14.3	71.8±14.5	60.2±10.5	0.032
Between groups (P2)	0.010	0.455	<0.001	
**Center (% of time)**				
CKO	23.8±9.0	25.4±8.3	21.9±10.1	0.279
CTL	31.9±14.3	28.2±14.5	39.8±10.6	
Between groups (P2)	0.009	0.457	<0.001	0.032
**Table 1B: (Open Field)**				
**N (number of measurements)**				
CKO	37	22	15	
CTL	32	22	10	
**Total distance (cm)**				
CKO	19832.3±6407.2	22077.7±6370.5	16539.1±4998.0	0.008
CTL	8896.1±2022.3	9384.3±2040.7	7790.1±1558.5	0.036
Between groups (P2)	<0.001	<0.001	0.002	
**Periphery (cm)**				
CKO	13526.6±4215.1	14877.0±4152.0	11545.9±3571.9	0.016
CTL	5762.4±1579.2	6226.3±1563.9	4742.0±1095.8	0.011
Between groups (P2)	<0.001	<0.001	<0.001	
**Center (cm)**				
CKO	6286.4±2714.7	7176.2±2953.2	4981.4±1673.4	0.014
CTL	3100.1±1158.0	3124.9±1308.6	3045.5±786.6	0.861
Between groups (P2)	<0.001	<0.001	0.002	
**Periphery (% of time)**				
CKO	74.9±11.4	74.0±13.7	76.3±7.06	0.557
CTL	68.2±14.5	69.9±15.7	64.4±13.1	0.348
Between groups (P2)	0.041	0.354	0.007	
**Center (% of time)**				
CKO	24.9±11.4	26.0±13.7	23.5±7.07	0.531
CTL	31.8±14.9	30.1±15.7	35.5±13.0	0.351
Between groups (P2)	0.039	0.356	0.006	

(A)- at 10 minutes; (B)- at 30 minutes. P1- represents independent samples T Test analysis comparing young and old animals in each group. P2– represents T Test analysis comparing CKO vs CTL.

### 2.3 The Elevated Plus Maze Test

The elevated plus maze (EPM) test consists of two open arms (25 × 5 cm, with 3-mm-high ledges) and two closed arms (25 × 5 cm, with 15-cm-high transparent walls) of the same size. The arms of the same type were arranged at opposite sides; all the arms and central squares were made of white plastic plates and elevated above the floor. Each mouse was placed in the central square of the maze (5 × 5 cm), facing one of the closed arms, and was recorded for 6 min (CKO, n=15; CTL, n=15; **Table 2**). Time spent in the closed or open arms or in the center (seconds), the proportion of time spent in open or closed arms (% time), the distance traveled (cm), and the number of total entries into the arms were calculated automatically by the EthoVision software. EPM is a behavioral test widely employed to assess anxiety-like behaviors in mice, relying on the aversion of rodents to open spaces (11).

**Table 2:** Comparison between CKO and CTL animals in the Elevated Plus Maze (EPM) test according to age group (data is presented as mean±SD).

	All	Young (<9 months)	Old (≥9 months)	Within group (P1)
**Table 2 (EPM test)**				
**N (number of mice)**				
CKO	15	8	7	
CTL	15	8	7	
**Total distance (cm)**				
CKO	1555.5±333.4	1596.1±297.4	1509.1±389.1	0.632
CTL	1153.1±224.0	1253.4±64.2	1038.4±289.0	0.098
Between groups (P2)	0.001	0.014	0.025	
**Open arms (cm)**				
CKO	231.6±145.6	199.1±137.0	268.7±156.7	0.375
CTL	285.1±135.0	341.9±93.6	220.2±151.9	0.080
Between groups (P2)	0.306	0.029	0.567	
**Open arms (% of time)**				
CKO	15.8±8.4	14.3±7.5	17.6±9.5	0.463
CTL	24.6±10.2	29.6±7.9	18.9±9.8	0.035
Between groups (P2)	0.016	0.001	0.815	
**Closed arms (cm)**				
CKO	921.8±297.6	999.7±284.5	832.7±307.9	0.295
CTL	608.7±146.8	658.2±118.3	552.1±164.1	0.171
Between groups (P2)	0.002	0.007	0.055	
**Closed arms (% of time)**				
CKO	49.5±16.9	53.8±18.0	44.6±15.3	0.310
CTL	49.3±12.7	48.1±11.5	50.7±14.8	0.706
Between groups (P2)	0.974	0.463	0.462	
**Center (cm)**				
CKO	402.1±105.2	397.2±125.3	407.6±86.2	0.856
CTL	259.3±69.9	253.3±56.8	266.1±86.7	0.739
Between groups (P2)	<0.001	0.015	0.010	
**Center (% of time)**				
CKO	34.7±13.7	31.9±14.1	37.8±13.5	0.426
CTL	26.1±8.0	22.3±5.8	30.4±8.3	0.044
Between groups (P2)	0.048	0.096	0.243	
**Entries –closed arms (n)**				
CKO	34.9±12.3	37.8±9.8	31.7±14.8	0.362
CTL	20.6±3.2	20.9±3.5	20.3±3.1	0.736
Between groups (P2)	<0.001	0.001	0.088	
**Entries –center (n)**				
CKO	49.6±11.8	51.4±9.8	47.6±14.2	0.552
CTL	33.5±5.3	34.4±3.1	32.6±7.2	0.555
Between groups (P2)	<0.001	0.001	0.028	
**Entries –open arms (n)**				
CKO	19.6±8.0	20.6±9.5	18.4±6.3	0.614
CTL	14.7±4.6	14.9±1.6	14.4±6.7	0.869
Between groups (P2)	0.050	0.135	0.273	

P1- represents independent samples T Test analysis comparing young and old animals in each group. P2- represents T Test analysis comparing CKO vs CTL.

### 2.4 Morris Water Maze Test

Spatial learning and memory were measured using the Morris water maze test ***(*** MWM) in a circular water pool (diameter – 1.2 m) filled with water (22 ± 1◦C). The water maze was divided into four quadrants according to the ‘+’ shape; and a transparent platform was placed in the center of the 1^st^ quadrant. The mice entered the water from the middle of the 1^st^, 2^nd^, 3^rd^ and 4^th^ quadrants, and the escape latency was recorded, namely the time from searching the platform to climbing the platform (CKO, n=15; CTL, n=15). If a platform was not found within 60 sec, the mouse was placed on the platform for 20 sec and then removed from the water maze, and the time was recorded as 60 sec. Each mouse was trained 4 times daily, and the mean time was calculated. The experiment was conducted for 6 consecutive days. A place navigation test was performed in the first 3 days, a space exploration test on the 4^th^ day, and a reversal phase test on the 5-6^th^ day. Space exploration was performed when the platform in the 1^st^ quadrant was removed, and the mice entered the water from the middle of the 2^nd^ quadrant. The traversing times, within 60 sec, across the target quadrant (i.e., the 1^st^ quadrant) and the swimming time were recorded. During the reversal phase, the platform was moved to another quarter of the maze and the time was recorded to reach the platform. Data were recorded using the automated EthoVision tracking system. The test assesses the ability of the animals to locate the underwater hidden platform, using surrounding visual cues; and is thus used to explore spatial memory and learning.

### 2.5 Voluntary Wheel Running

Mice were allowed to run freely on the open surface of a slanted plastic saucer-shaped wheel [Low-Profile Wireless Running Wheel (Med Associates, Fairfax, USA) for mouse (15.25 × 10.25 × 3.3 cm)] placed inside the mouse cage, within routine daily rhythmicity patterns in a non-stressed laboratory environment (CKO, n=15; CTL, n=14). Rotations were electronically transmitted to a USB hub (DIG-804 USB interface hub), such that the frequency and rate of running could be captured. Mice were individually housed so that accurate recordings could be made for each animal. The hourly sum of wheel revolutions (wheel spins) was collected by SOF-860-wheel manager software (Med Associates, Georgia, USA). Data were collected 6 days, 24 h a day (12, 13). The pattern of running during day (light time; L) and night (dark time; D) was analyzed and used to measure voluntary activity.

### 2.6 Serum Analysis of Uncarboxylated Osteocalcin (Glu- Osteocalcin)

Uncarboxylated osteocalcin (GluOC) was measured, since previous work showed that it may connect bone and brain function, especially anxiety (14). Serum samples (CKO, n=26; CTL, n=22) were analyzed using an Elisa kit (Cat # MK129; Takara, Japan) according to the manufacturers’ instructions (detection limits=0.25ng/ml).

### 2.7 Western Blot Analysis of SIRT1

Brains of CKO and CTL mice were homogenized in a lysis buffer (200mM HEPES, 5mM EDTA, 150mM NaCl, 1% NP-40, 0.5% Na-deoxycholate), which was supplemented with a protease inhibitor cocktail (Roche, Basel, Switzerland) in a 1:12 ratio and phosphatase inhibitor cocktail (Roche, Basel, Switzerland) in a 1:12 ratio and phosphatase inhibitor cocktail (Roche, Basel, Switzerland) in a 1:10 ratio. Protein concentration was determined using the Pierce BCA Protein Assay Kit (Thermo Scientific, IL, USA), and 100μg proteins were analyzed per sample by sodium dodecyl sulfate (SDS)-polyacrylamide gel electrophoresis (PAGE), followed by western immunoblotting. Proteins were transferred to nitrocellulose membranes (GE Healthcare, NJ, USA) by the wet blotting system (Bio-Rad, Hercules, CA, USA). Nitrocellulose membranes were then incubated in TBS-T (10mM Tris-HCl, 150mM NaCl, 0.1% Tween 20) with 5% skim milk solution for one hour to block nonspecific binding, and then incubated with the primary antibody (anti Sirt1- Millipore Cat #07-131) overnight at 4°C. Membranes were washed with TBS-T, incubated with a secondary fluorescent antibody (LI-COR Biosciences, Lincoln, NE, USA) decorated with IRDye^®^ for one hour and washed again. Alpha-Tubulin (Cat#2144 Cell Signaling) served as the reference. Quantification was performed using the Odyssey (2.1.) application Software (LI-COR Biosciences, Lincoln, NE, USA)

### 2.8 Statistical Analysis

***2.8.1*** A mixed-model repeated-measures analysis was conducted to compare the effect of group (CTL vs. CKO) on weight during the study period. This analysis enabled using all the available data from the full sample of randomized animals, without imputing missing values. The model was specified with a between-group factor (CTL vs. CKO), a within-group factor of time, and group x time interaction. For this analysis, data are expressed as estimated marginal means and standard error.

***2.8.2*** For the between group comparison of the behavioral test results (OFT, EPM, MWM), independent sample T-tests were conducted.

***2.8.3*** For the MWM test analyses, a Cox regression analysis was used to evaluate the effects of the group (CKO vs. CTL) and the animal age on the time until test success for each of the experiment days.

***2.8.4*** For the running wheels test, area under the curve (AUC) was calculated for each period of time and compared between the groups using Independent Samples Mann Whitney U tests (due to skewed distribution of the AUC).

***2.8.5*** Data were generated and analyzed with SPSS software, version 25.0 (IBM). Differences were considered statistically significant at P<0.05.

## 3 Results

***3.1. Effect on body weight***: CKO mice were generally heavier (**Supplementary Figure 1**), with no significant differences in food consumption (average food consumption 3.68 ± 0.04 gr/day for the CTL mice and 3.56 ± 0.3 gr/day for the CKO mice; p=0.27). At the age of 21 days, no significant differences in weight were noted, however, there was a significant difference in the change over time between the CKO and CTL mice (β=0.03, SE=0.01, P=0.006), with a greater increase in weight gain in the CKO group [CTL group: y (weight) =14.92+0.16X (time); CKO group: y=14.08+0.19X] (2). We did not notice differences between the groups in behavior when they were in their home cages, either housed in small groups or in solitary cages (2).

**Figure 1:**
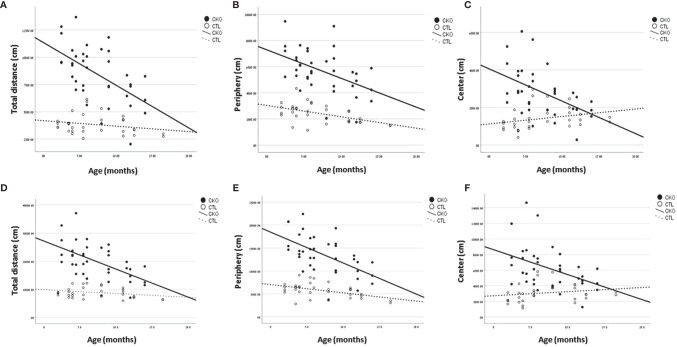
Results of the open field test (OFT). Graphs showing, for each of the groups (CKO and CTL), correlations between the animal age and **(A, D)** The total distance covered, **(B, E)** distance travelled in peripheral zone, **(C, F)** distance travelled in central zone. **(A–C)**- 10 minutes; **(D–F)** 30 minutes.

***3.2. Open field test (OFT) -*** using the OFT, no differences in gait were observed, however, there were significant differences in the number of steps (i.e. distance travelled). The results are presented in **Table 1** and **Figure 1** the first 10 minutes are presented in **Table 1A** and **Figures 1A–C**; results of the whole test (30 min.) are presented in **Table 1B** and **Figures 1D–F**. The distance covered by the CKO mice was substantially greater than that covered by the CTL mice, in both the first 10 minutes and when calculated for the whole duration of the test (in both P<0.001) (**Table 1**). While the effect of age was not significant in the first 10 minutes, the distance travelled during the whole period of the test (30 min.) decreased with age, in both CKO and CTL mice. Even though the distance was reduced with increased age of the CKO mice it was still significantly greater than in the CTL group (young CKO vs young CTL; Old CKO vs Old CTL, P<0.005 for both) (**Table 1**). CKO mice spent more time in the periphery and less in the center compared to CTL mice (p<0.05). To examine if greater anxiety might explain these differences, we performed a test that is more specific for anxiety assessment, the elevated plus maze test.

**Figure 2:**
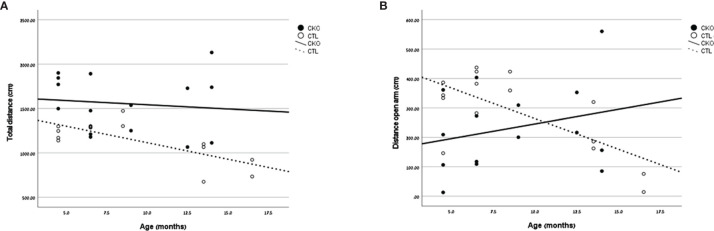
Results of the elevated Plus Maze. Graphs showing, for each of the groups (CKO and CTL), correlations between the animal age and **(A)** total distance covered (cm) or **(B)** distance covered in the open arms (cm).

***3.3. The elevated plus maze (EPM)***- In the EPM test (**Table 2** and **Figure 2**), the CKO mice covered a significantly greater distance than did the CTL mice (P<0.001); the difference was significant for both younger (P=0.014) and older mice (P=0.025) (**Figure 2A**). The time spent in the open arms was significantly less in young CKO than in young CTL (P=0.001). However, the time was similar in the older age group (P=0.815). This was evident by less time spent in the open arms among older than younger CTL mice (P=0.035), yet no such difference by age was observed among the CKO mice (**Figure 2B**). The number of entries into all the sections of the maze were greater for CKO than CTL mice (P ≤ 0.05). This indicates that the CKO mice were moving about quickly, although differences from the CTL were statistically significant only in entries by younger CKO mice into the closed arms and the center (P=0.001), and in entries of older CKO mice into the center (P=0.028). Taken together, these results support the greater anxiety of CKO mice that was demonstrated in the OFT.

**Figure 3:**
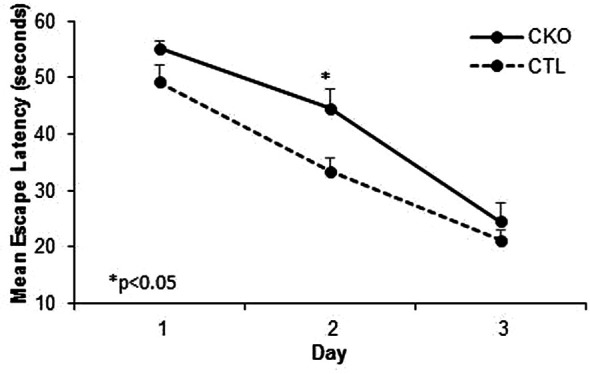
Results of the Morris water maze (MWM). Cumulative escape latency in all 3 days comparing CKO and CTL mice. Data are presented as mean ± SD, *P < 0.05.

***3.4. Morris water maze***. We next compared spatial memory and learning abilities of CKO to CTL mice using the Morris water maze (**Figure 3** and **Table 3**). The study was conducted for six consecutive days. Both groups showed improved learning with time, and on the third day, all the animals (from both groups) reached the platform rapidly, with no significant differences between the groups. In the first three days an improvement was noted for both CKO and CTL mice, however, CKO mice were slower in finding the platform on the first (P=0.076) and second (P=0.014) days. Comprehensive Cox regression analysis for the first 3 days (**Table 3**) supported these results. No differences were observed between the CKO and CTL mice in the space exploration and the reversal phase tests (days 4-6; data not shown).

**Table 3:** Multivariable Cox regression analysis for the MWM tests (time to platform) during the first three days.

Groups	Day	Point Estimate	95% Confidence Limits	Z Value	P
**Table 3 (MWM test)**					
CKO *vs* CTL	1	0.526	0.146-1.90	-0.98	0.327
CKO *vs* CTL	2	0.139	0.040-0.486	-3.09	<0.002
CKO *vs* CTL	3	0.577	0.177-1.884	-0.91	0.362
CKO	1 *vs* 2	0.223	0.085-0.584	-3.05	0.0023
CKO	2 *vs* 3	0.050	0.018-0.139	-5.74	<0.0001
CKO	1 *vs* 3	0.011	0.003-0.036	-7.51	<0.0001
CTL	1 *vs* 2	0.059	0.021-0.161	-5.50	<0.0001
CTL	2 *vs* 3	0.207	0.087-0.491	-3.57	0.0004
CTL	1 *vs* 3	0.012	0.004-0.040	-7.23	<0.0001
CKO young	1 *vs* 2	0.188	0.049-0.727	-2.42	0.015
CKO young	2 *vs* 3	0.019	0.005-0.077	-5.52	<0.0001
CKO young	1 *vs* 3	0.004	0.001-0.018	-6.8	<0.0001
CTL young	1 *vs* 2	0.06	0.017-0.218	-4.28	<0.0001
CTL young	2 *vs* 3	0.221	0.068-0.721	-2.5	0.0123
CTL young	1 *vs* 3	0.013	0.003-0.06	-5.6	<0.0001
CKO old	1 *vs* 2	0.174	0.042-0.714	-2.43	0.0152
CKO old	2 *vs* 3	0.086	0.022-0.333	-3.55	0.0004
CKO old	1 *vs* 3	0.015	0.003-0.066	-5.52	<0.0001
CTL old	1 *vs* 2	0.035	0.008-0.146	-4.60	<0.0001
CTL old	2 *vs* 3	0.161	0.049-0.534	-2.99	0.0028
CTL old	1 *vs* 3	0.006	0.001-0.028	-6.32	<0.0001
CKO young *vs* CKO old	1	1.61	0.225-11.497	0.47	0.635
CKO young *vs* CKO old	2	1.745	0.268-11.364	0.58	0.56
CKO young *vs* CKO old	3	0.378	0.062-2.309	-1.05	0.292
CTL young *vs* CTL old	1	0.644	0.092-4.483	-0.44	0.6566
CTL young *vs* CTL old	2	1.106	0.194-6.306	0.11	0.9093
CTL young *vs* CTL old	3	1.513	0.252-9.092	0.45	0.651
CKO young *vs* CTL young	1	0.303	0.044-2.079	-1.22	0.2242
CKO young *vs* CTL young	2	0.097	0.016-0.593	-2.53	0.0115
CKO young *vs* CTL young	3	1.15	0.20-6.614	0.16	0.875
CKO old *vs* CTL old	1	0.757	0.103-5.554	-0.27	0.7841
CKO old *vs* CTL old	2	0.153	0.022-1.045	-1.92	0.0555
CKO old *vs* CTL old	3	0.288	0.044-1.87	-1.3	0.192

CKO, collagen type II-specific Sirt1 knockout mice; CTL, Sirt1 flox/flox control mice without the Cre transgene.

These analyses clearly show that there is a significant difference between the groups on day 2 of the MWM, while no significant difference is found between young and old mice in both groups.

**Figure 4:**
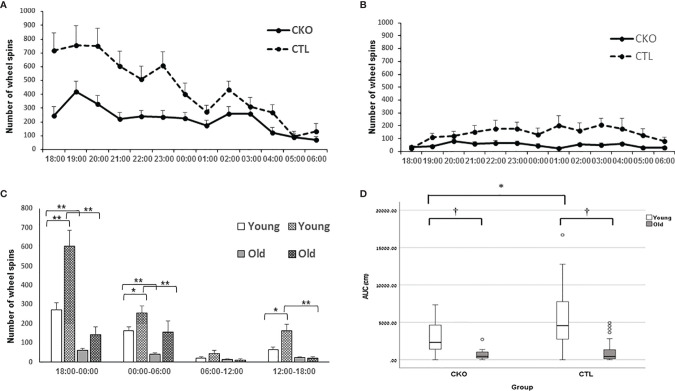
Results of the free running wheels. The average sum of hourly wheel revolutions (wheel spins) of **(A)** young and **(B)** old mice old during the night cycle. **(C)** Hourly sum of wheel revolution according to 6 hours interval during the day and night (*p < 0.05; **p = 0.001). **(D)** AUC analysis of the hourly sum of wheel spins of the active period (night) presented in **A** and **B** (*p < 0.05; †< 0.01).

***3.5. Free running wheels.*** Wheel running in the home cages was used to assess voluntary activity in contrast to other experimental platforms that we used. After mice were allowed to accustom to the free running wheels, their running activity was recorded. Both the CKO and CTL groups used the wheels mostly during the active dark period (**Figures 4A–C**). The young mice showed significantly greater activity than the older mice in both groups (P<0.001), both in the night and the day (**Figure 4C**). However, contrasting with the results of the OFT and the EPM, young CKO mice showed significantly reduced activity compared to the CTL mice (P=0.001) (**Figures 4A–D**). No differences in circadian period were observed (CTL 24.4 hours ± 0.88, CKO 24.0 ± 1.48; P=0.5).

***3.6. Osteocalcin levels:*** Uncarboxylated osteocalcin (GluOC) was measured, since previous work showed that it may connect bone and brain function, especially anxiety (14). Levels of GluOC tended to be higher in the young CKO mice than in the young CTL mice, but the difference in magnitude was small (CKO 16.4 ± 1.3 ng/ml, CTL 15.0 ± 0.7 ng/ml; P=0.06). Levels were significantly reduced with age in both groups; differences between the CKO and CTL mice at age 3 months or older were not statistically significant.

**Figure 5:**
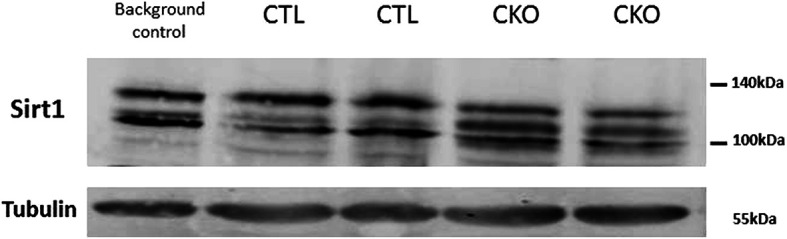
Results of the western blot analysis of SIRT1. Significant differences in the pattern of SIRT1 in protein extracts derived from brains of C57BL mice (background control), CTL mice, CKO mice. The relative distribution of the different forms, in extracts derived from CTL mice is identical to that of other control mice (C57BL) and different from that of CKO mice.

***3.7. Levels of* SIRT1 *in the brains*** of CKO and CTL mice were visualized using a western blot analysis, as several studies in mouse embryogenesis (9, 15–17), showed transient expression of Col type II mRNAs in the brain. No gross difference in the total amount of SIRT1 protein was observed. Three bands with apparent molecular weight of 100-130 kDa were observed per lane, a pattern that is commonly seen in samples taken from cytosols or whole cells, compared to a single band of 110 kDa detected in nuclear extracts (18). In extracts derived from the CTL mice, the pattern was identical to that of a control mouse (C57Bl/6, which are the most widely used genetic background for genetically modified mice). In contrast, the relative distributions of the various bands differed substantially between the CKO and CTL mice; with the intermediate band more pronounced in extracts of CKO compared to CTL (**Figure 5**).

## 4 Discussion

In this study, we found that Sirt1 knockout (CKO) mice have reduced activity, increased anxiety and decreased spatial learning and memory, compared to CTL mice. In a previous study (8), we found that the transgenic CKO mice had shorter bones with less organized EGP, had increased weight, reduced bone mineralization and were less responsive to the nutritional manipulation, with less efficient CUG.

While no gross effect was found in their gait, the CKO mice covered a significantly greater distance than the CTL mice in both the OF (19) and EPM tests (20, 21), and spent relatively more time in the sheltered areas of the arena or the maze, respectively. Increased levels of anxiety usually lead to less locomotion and decreased exploratory behavior with a preference to stay close to the walls of the field; while in this case the animals showed increased activity with decreased exploratory behavior. In contrast to the increased activity in the above- mentioned tests, the use of home cage running wheels showed reduced activity. This indicates that the increased locomotion activity of the CKO mice in the OF and the EPM tests was due to increased anxiety, and the increased body weight we noted at the initiation of this research can be attributed to a lower level of activity in the home cage. Slower learning revealed by the MWM, was evident during the first two days of the tests (especially on the second day). Data from the home cage running wheels and from circadian rhythm analysis indicated that there was no significant difference between the groups in the circadian period, this is in contrast to the brain-specific SIRT1 knockout mice (BSKO) (22, 23) that showed elongated circadian period (24–26).

Compared to younger mice, older CTL tended to be less cautious in the OFT, exploring more willingly the center of the arena, consistent with previous reports of mice of a similar genetic background, C57BL/6J  (27, 28); no such effect was observed for old CKO mice. On EPM, maybe due to the shorter duration of the test, there was no significant effect of age on the total distance traveled in both groups. However, old CTL spent more time in the center compared to young CTL (P=0.044), concomitant with a reduction in the % of time spent in the open arms (p=0.035), and similarly to the OFT, no such effect was noted in the CKO mice.

Taken together, our results indicate that the CKO mice entailed disruption in a central control mechanism that affects activity, anxiety and cognition. Since we used a common system of Cre/Col II, expected to be specific for cartilage, the explanation for the effect on cognitive function and behavior was elusive. An extensive review of the literature suggests several possible explanations.

One is that the knockout of SIRT1 from EGP cartilage, which affected bone mineralization and maturation (2), affected the level of osteocalcin (OC) and its un-carboxylated form (GluOC); the latter was shown to serve as a link between bone and brain function, especially anxiety (14). OC is expressed and secreted by osteoblasts, hypertrophic chondrocytes, and adipocytes (29), and is the most abundant non-collagenous protein in bone. OC was found to be involved in multiple physiological processes such as energy metabolism, adipogenesis, neuronal development, muscle growth, and male fertility (30). Despite the important role of OC in bone mineralization and calcium ion homeostasis, OC −/− mice showed only moderately increased bone mass (31). Interestingly, however, these mice showed unexpectedly increased anxiety (31). OC was later found to regulate proper brain development and function, and OC−/− mice had smaller and less developed brains. The mature OC is post-translationally modified on three glutamate residues; a process that increases the affinity of OC for hydroxyapatite crystal; thus, most secreted OC is embedded in the bone matrix. The acidic environment generated during bone resorption promotes decarboxylation of OC to GluOC, decreasing its affinity and promoting its release into the circulation. GluOC crosses the blood brain barrier, directly preventing anxiety and depressive behavior while also strengthening spatial learning and memory. Therefore, if GluOC activity would explain the findings of the current study, we would have expected to see lower levels of GluOC in the serum of CKO mice. Serum analysis showed that the levels of GluOC were significantly reduced with age in both groups as also reported by others (32), however, we found a small increase in GluOC levels in CKO mice, which tended to be significant only in young animals. This may be associated with the reduced bone mineralization in CKO mice (2). In addition, our mice mated and reproduced normally, with no difference between the groups, also supporting the finding that GluOC levels were comparable between the groups (30). Our findings contrast with a publication that reported an inverse association between GluOC and body weight (33). We therefore conclude that GluOC does not explain the increased anxiety and lower cognitive function in the CKO mice.

Another plausible explanation is that the Cre-Col II system affected brain SIRT1, as effects similar to those we see in learning, memory and anxiety were previously reported in brain-specific SIRT1 knockout mice (BSKO) (22, 23), this is in spite of the fact that the driver that we used for Cre was Col type II and not nestin (34). Indeed, a recent review summarized the off-target effect of many skeletal Cre lines, showing that most Cre lines show some level of unintended activity in other tissues (35), thus the notion that Cre lines are specific to a particular cell or tissue has been misleading. While collagen type II is usually considered a hallmark for chondrogenic differentiation, several publications on mouse embryogenesis (9, 15–17), as well as a study on the Cre Col II system (36) showed transient expression of Col type II mRNAs in a number of non-chondrogenic tissues such as notochord, sclerotome, pre-chondrogenic mesenchyme, heart, brain and eye. This transient expression (16), is suggested to have an important role in the proper development of all brain structures (17). The early expression of the collagen type II gene in the neuroepithelium at 9.5 days coincides with the period of extensive morphogenesis of the neural tube (15).

In BSKO it was shown that loss of function of SIRT1 impairs synaptic plasticity, memory formation and spatial learning, probably *via* a microRNA-mediated mechanism (22). SIRT1 has been suggested as a component of the molecular pathways that determine the fate of neuronal progenitor cells (37). Analysis of SIRT1 in the brain has shown that *Sirt1* mRNA is highly expressed in metabolically relevant sites, including, the hypothalamic arcuate, and some areas in the hindbrain (38). In addition, it was suggested that SIRT1 regulates anxiety by the de-acetylation of a transcription factor regulating the monoamine oxidase A gene in the brain, which is involved in oxidative deamination of dopamine, norepinephrine, and serotonin (39). Furthermore, in the same model, SIRT1 was shown to be involved in regulating the circadian rhythm (25).

There is a significant overlap between the expression of SIRT1 and sites of embryonic expression of collagen type II. However, western blot analysis of brain SIRT1 did not show the expected reduction in Sirt1 protein level, unlike the reduction shown in cartilage (2); in contrast, we found a significant difference in the pattern of SIRT1 forms. This may indicate changes in post-translational modifications, such as phosphorylation, as SIRT1 is known to be phosphorylated on numerous sites (40). Phosphorylation can increase its nuclear deacetylase activity (41, 42), and may also induce SIRT1 ubiquitination and proteasomal degradation (41).

It is important to note that there are several discrepancies between our findings and those of BSKO. For example, while it was reported that hypothalamic SIRT1 is crucially important for the central regulation of food intake (43), in our mice there was no difference in food consumption.

Furthermore, CKO mice showed hypo-activity when in their home cage, while brain POMC- SIRT1 k/o showed unaltered levels of activities (44). BSKO mice were reported to be dwarf (34), had reduced GH secretion, and reduced body weight and length at 10 weeks while in our study, CKO mice did not show gross growth inhibition; in contrast, their body weight was increased. In addition, BSKO mice were reported to have elongated circadian period, while the CKO and CTL show similar circadian period (26, 45–47)

Overall, it is still not clear what are the underlying causes for the differential behavior of the CKO mice. Some of the behavioral presentation of the CKO mice are reminiscent of Attention Deficient Hyperactive Disorder (ADHD), in their reduced sensory regulation. Interestingly, a recent publication on children with ADHD showed significantly lower SIRT1 levels and significantly higher metalloproteinase-9 (MMP-9) levels in the serum (48). MMP-9, an endopeptidases involved in degradation of the extracellular matrix, is negatively regulated by SIRT1; MMP-9 and SIRT1 both act in the EGP and the brain and are important for brain development, synaptic plasticity, learning and memory (49). Their presence in the serum may suggest another link between EGP and brain.

Two thirds of the genetic syndromes with short stature as a feature listed in the genetics database, POSSUM (https://www.possum.net.au/) are associated with intellectual disability. Furthermore, more children with idiopathic short stature (ISS) were shown to have a lower IQ, lower fluid and quantitative reasoning, and substantial deficits in the visual-motor skills and working memory than healthy controls (50).

One possible explanation for lower test scores in intelligence and academic achievement tests in children with ISS might be an underlying condition that has caused both short stature and cognitive impairment, such as a genetic cause, malnutrition, improper psychosocial environment, the presence of general diseases and specific hormonal deficits. The influence of the environmental factors may be intertwined, making it difficult to ascertain the role of a specific factor in humans. Despite the significant progress in unraveling the complex mechanisms required for proper growth and development, the therapeutic options for both conditions are extremely limited. It has been proposed that pharmacological activation of SIRT1 with the SIRT1 activator, resveratrol, or with synthetic SIRT1-activating compounds (i.e.SRT2104) are used to treat neuropsychiatric disorders (51). Maybe these should be also considered for use in children with growth and development disorders, as we have previously shown that SIRT1 is required for efficient CUG (2). However, one should bear in mind that these studies were performed in a mice model and conclusions from this model to developmental process in children should be drawn with utmost caution.

Several publications show that SIRT1 plays an important role also in puberty, at least in females; over expression of SIRT1 in the hypothalamus was shown to cause delayed puberty in female mice (46, 47). Thus, in the current study, if the underlying cause for the behavioral changes had been due to reduction of SIRT1 in the brain, mice would have been expected to present with precocious puberty. No gross effect differences were noted in either male or females, although we did not check testosterone in males nor vaginal opening in the females, and this may be a limitation of the study (52). However, as most tests were performed on post pubertal mice, we do not consider it to be a significant confounding factor. Another limitation concerns the western blot, in which we have no explanation as to the different pattern of brain SIRT1 protein; it would have been best if we could have compared the brains to those of BSKO, however we could not obtain these mice.

The nature of the mechanism that enables the connection between growth and behavior is still elusive; we hope that in the future we will be able to decipher the underlying cause, and maybe find a common regulator that affects growth and cognition; this may open a new area for intervention and will enable development of novel therapeutic modalities.

## Data Availability Statement

The raw data supporting the conclusions of this article will be made available by the authors, without undue reservation.

## Ethics Statement

The animal study was reviewed and approved by Institutional Animal Care and Use Committee of Tel Aviv University, that follows the NIH guide for the care and us of laboratory animals (protocol approval number 01-16-052) prior to onset of the study.

## Author Contributions

BS: conducted the study, analyzed the data and prepared the tables and figures. SHH helped performing and analyzing behavioral tests. MP: funding acquisition, MY-G performed the statistical analysis. GG-Y: conceived the idea, supervised the study, analyzed the data and wrote the paper. All authors contributed to the article and approved the submitted version.

## Conflict of Interest

The authors declare that the research was conducted in the absence of any commercial or financial relationships that could be construed as a potential conflict of interest.

## Publisher’s Note

All claims expressed in this article are solely those of the authors and do not necessarily represent those of their affiliated organizations, or those of the publisher, the editors and the reviewers. Any product that may be evaluated in this article, or claim that may be made by its manufacturer, is not guaranteed or endorsed by the publisher.

## Supplementary Material

Supplementary Figure 1 Follow-up of body weight to age 88 days shows that the CKO mice are generally heavier than the CTL mice (re-published with permission obtained from Bone).

## Abbreviations

CKO, collagen type II-specific Sirt1 knockout; CTL, control; Sirt1, sirtuin 1; OC, osteocalcin.
