# Supplementary figures and images for: Anxiety and Cognition in Cre- Collagen Type II Sirt1 K/O Male Mice
Source: Front Endocrinol (Lausanne). 2021 Nov 19;12:756909. doi: 10.3389/fendo.2021.756909 (PMC8641514; doi:10.3389/fendo.2021.756909)

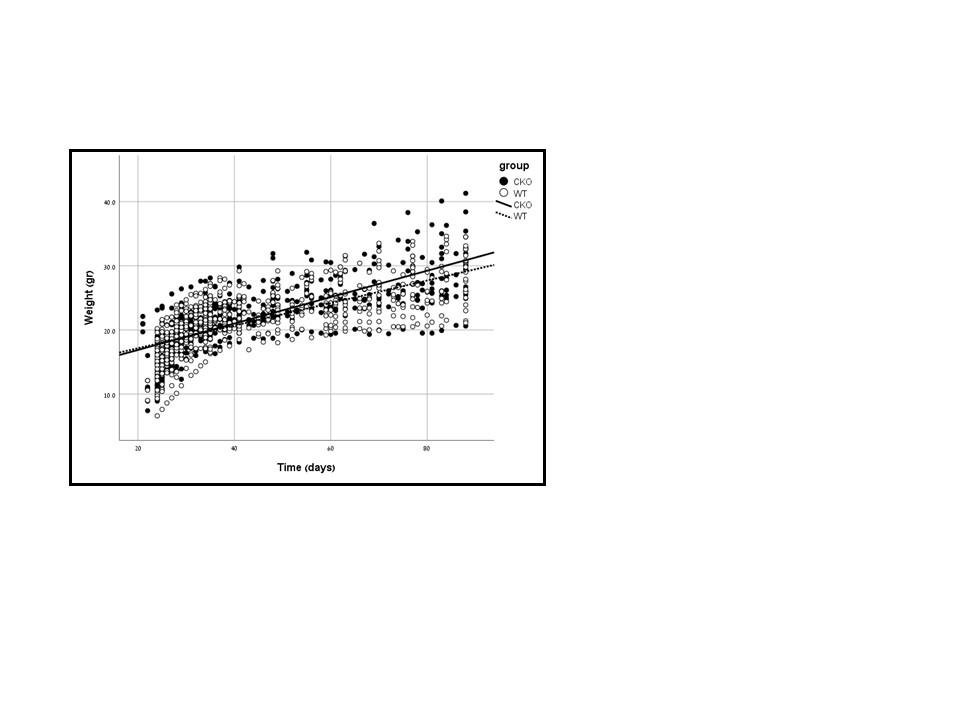

Supplement: Supplementary Figure 1 — Follow-up of body weight to age 88 days shows that the CKO mice are generally heavier than the CTL mice (re-published with permission obtained from Bone). [file Image_1.jpeg]
